# A phase I study of the human anti‐activin receptor‐like kinase 1 antibody PF‐03446962 in Asian patients with advanced solid tumors

**DOI:** 10.1002/cam4.724

**Published:** 2016-04-14

**Authors:** Toshihiko Doi, Kyung‐Hun Lee, Tae‐Min Kim, Atsushi Ohtsu, Tae Yong Kim, Masafumi Ikeda, Kiyotaka Yoh, Corrado Gallo Stampino, Tomoko Hirohashi, Akiyuki Suzuki, Yosuke Fujii, James Andrew Williams, Yung‐Jue Bang

**Affiliations:** ^1^ National Cancer Center Hospital East6‐5‐1 KashiwanohaKashiwaChibaJapan; ^2^ Seoul National University College of Medicine and Hospital101 Daehak‐roJongno‐guSeoul110‐744South Korea; ^3^ Pfizer Global Oncology Research and DevelopmentVia Anna Maria Mozzoni, 12Milan20152Italy; ^4^ Pfizer Japan3‐22‐7 YoyogiShibuya‐kuTokyo151‐8589Japan; ^5^ Pfizer Oncology10555 Science Center DriveSan Diego92121California

**Keywords:** Activin receptor‐like kinase 1, bone morphogenetic protein, hepatocellular carcinoma, PF‐03446962, solid tumors

## Abstract

Preclinical studies suggest that ALK‐1 signaling mediates a complementary angiogenesis pathway activated upon development of resistance to vascular endothelial growth factor (VEGF)‐targeted therapies. Inhibition of ALK‐1 signaling may lead to disruption of tumor angiogenesis and growth. We report findings from a multicenter, open‐label, phase I study of the fully human anti‐ALK‐1 mAb PF‐03446962 conducted in Japan and South Korea, in Asian patients with advanced solid tumors. The dose escalation Part 1 of the study was based on a standard 3 + 3 design (*n* = 16). In Part 2, patients were treated with PF‐03446962 at 7 and 10 mg/kg (10/cohort), including patients with disease progression following prior VEGF receptor (R)‐targeted therapy. Primary objectives were determination of the maximum tolerated dose (MTD) and recommended phase II dose (RP2D). Secondary objectives included safety, pharmacokinetics, pharmacodynamics, and antitumor activity of PF‐03446962. No dose‐limiting toxicity (DLT) was noted in the 12 DLT‐evaluable patients. Treatment was well tolerated. The MTD for biweekly intravenous administration was estimated to be 10 mg/kg and the RP2D 7 mg/kg. Treatment‐related grades 1–3 thrombocytopenia was experienced by 27.8% patients. The most frequent nonhematologic treatment‐related AEs were grades 1–2 pyrexia and epistaxis. Four patients (3/4 with hepatocellular carcinoma) developed telangiectasia suggesting vascular targeting and in vivo ALK‐1 inhibition by PF‐03446962. Stable disease for 12 weeks or more was observed in 25.7% of patients and in 44.4% of those with hepatocellular carcinoma. ALK‐1 inhibition by PF‐03446962 may represent a novel antiangiogenic strategy for patients with advanced solid malignancies complementary to current treatment with VEGF(R)‐targeted inhibitors or chemotherapy.

## Introduction

The activin receptor‐like kinase‐1 (ALK‐1) is a type‐I transforming growth factor (TGF)‐*β* serine/threonine kinase receptor, preferentially expressed on proliferating endothelial cells in blood and lymphatic vessels. It binds the bone morphogenetic proteins (BMP)‐9 and 10, which are members of the transforming growth factor‐beta (TGF‐*β*) ligand super family 1, 2, 3, 4. Activation of ALK‐1, induced by ligand binding and formation of a membrane complex with TGF‐*β* and its type‐II receptor endoglin, leads to recruitment and phosphorylation of SMADs 1, 5, and 8, intracellular signaling, and modulation of target gene expression 4, 5.

Activin receptor‐like kinase‐1 plays a key role in the development of vessel networks, as demonstrated in type‐2 hereditary hemorrhagic telangiectasia (HHT) (Osler–Weber–Rendu syndrome), which is a disease characterized by loss‐of‐function mutations in the *ACVRL1* gene encoding for ALK‐1 and by abnormal vessel development (e.g., vascular dysplasia syndrome and arterial venous malformations) 6, 7, 8.

Activation of the ALK‐1/endoglin complex by BMP‐9/TGF‐*β* ligand binding has proangiogenic effects in tumors, as demonstrated in preclinical models, by induction of endothelial cell proliferation, migration, and tube formation 9, 10. Furthermore, signaling through the ALK‐1 pathway may represent one of the mechanisms allowing tumor escape from the inhibitory effects of vascular endothelial growth factor (VEGF)‐targeted therapies 11, 12. Consistent with a key function of the ALK‐1/endoglin complex in tumor vasculature, a longer overall survival has been reported in patients affected by HHT who developed breast, prostate, colorectal, or lung cancer. In particular, a diagnosis of HHT was found to be associated with a significantly better prognosis in patients with breast cancer 13.

PF‐03446962 is a fully human anti‐ALK‐1 mAb (IgG2) which has been shown to inhibit angiogenesis induced by proangiogenic factors such as VEGF‐A and basic fibroblast growth factor in Matrigel assays. PF‐03446962 also inhibited tumor growth in human xenograft models, by blocking angiogenesis in tumor‐associated blood and lymphatic vessels and reducing blood flow in mature vessels 12, 14, 15. In addition, preclinical studies have shown that PF‐03446962 inhibited ALK‐1 signaling, but did not interfere with the effects produced by VEGF in endothelial cells 15.

PF‐03446962 has demonstrated a favorable safety profile and preliminary evidence of antitumor activity in a phase I, first‐in‐human study conducted in Western patients with advanced solid malignancies 16. Responses were also noted in patients who had progressed after prior treatment with sorafenib and other VEGF receptor (VEGFR)–targeted antiangiogenesis therapies. These findings suggest that ALK‐1 signaling may represent a complementary angiogenesis pathway that can be activated upon development of VEGF resistance 17, 18. No antitumor activity was observed with single‐agent PF‐03446962 in patients with treatment‐refractory urothelial cancers who had received a median of three prior drugs 19. This phase I study was undertaken to estimate the maximum tolerated dose (MTD) and define the recommended phase II dose (RP2D) of PF‐03446962, and characterize safety, pharmacokinetics (PK), pharmacodynamic profile, and preliminary antitumor activity of PF‐03446962 in Asian patients with advanced solid tumors.

## Patients and Methods

### Study design and patient selection

This international, open‐label, single‐arm, phase I study was conducted in Asian patients with advanced solid tumors in Japan and South Korea. It was divided into two parts: dose escalation (Part 1) based on a standard 3 + 3 design and an expansion part with two cohorts (Part 2). Two dose‐level cohorts were to be selected for Part 2 based on the safety findings obtained in the dose escalation phase. Primary objectives of the study were to determine the MTD and the RP2D for treatment with PF‐03446962 in Asian patients with advanced solid tumors. Secondary objectives included the safety, PK profile, immunogenicity, pharmacodynamic effects, and preliminary antitumor activity of PF‐03446962, example, best overall response, clinical benefit rate, and progression‐free survival (PFS) in this patient population.

Patients with a histologically or cytologically confirmed diagnosis of locally advanced or metastatic solid tumors and refractory disease, intolerance to treatment, or no available standard therapy were included in Part 1 of the study. For enrollment in the Part 2 expansion cohorts, patients with advanced solid tumors, including hepatocellular carcinoma (HCC), had to have measurable lesions and disease progression following prior treatment with a VEGFR inhibitor or intolerance to available therapies. In addition, patients with HCC had to have total bilirubin ≤2.0 mg/dL, serum albumin ≥2.8 g/dL, and Child‐Pugh Class A or B. In both Parts 1 and 2, patients had to have Eastern Cooperative Oncology Group performance status (ECOG PS) of 0 or 1 and adequate bone marrow, renal, and hepatic functions.

Patients were excluded from the study if they had received chemotherapy, radiation therapy, or other investigational anticancer drugs within 4 weeks of study‐treatment initiation. In addition, patients were not eligible if they had active bleeding disorders, a corrected QTc interval >470 msec, a history of serious cardiovascular events in the prior 12 months, uncontrolled hypertension, HHT, or experienced excessive toxicities due to prior treatments.

The study was conducted in compliance with the Declaration of Helsinki and followed the International Conference on Harmonization Good Clinical Practices guidelines. The protocol was approved by the institutional review boards of the participating institutions and all patients provided signed informed consent. The study was supported by Pfizer and registered at ClinicalTrials.gov (NCT01337050).

### Treatment and dose‐limiting toxicity

In Part 1, PF‐03446962 was administered as a 1‐h i.v. infusion on days 1 and 29, and then once every 2 weeks, at a starting dose of 4.5 mg/kg. The dose level was escalated to 7 and 10 mg/kg using a 3 + 3 study design. In Part 2, patients received PF‐03446962 at 7 and 10 mg/kg (10 patients/dose level), based on the findings of the dose escalation part of the study. Treatment was continued until disease progression, patient withdrawal, or unacceptable toxicity.

Dose‐limiting toxicity (DLT) was defined as non–disease‐related adverse events (AEs) observed in the first 6 weeks of treatment that were possibly attributable to PF‐03446962, including grade 4 neutropenia (<500/mm^3^) for ≥8 days (not considered a DLT if resolved to grade ≤3 within 7 days), grade ≥3 febrile neutropenia, grade ≥3 neutropenic infection, grade 4 thrombocytopenia (<25,000/mm^3^), grade 3 thrombocytopenia (<50,000/mm^3^) with active bleeding, or grade ≥3 nonhematologic toxicity. However, grades 3–4 nausea, vomiting, and diarrhea controlled with antiemetics and antidiarrheal medications; grade 3 hypertension controlled with standard antihypertensive therapy; and nonclinically significant grades 3–4 electrolyte abnormalities were not to be considered DLTs.

### Assessments

#### Safety

Adverse events were monitored from day 1 of treatment until at least 28 days after the last dose, and graded for severity using NCI Common Terminology Criteria for Adverse Events, version 4.0.

#### Pharmacokinetics

Pharmacokinetics parameters for PF‐03446962 were evaluated in this study, including the maximum serum concentration, time to maximum serum concentration (*T*_max_), area under the serum concentration–time curve (AUC) to 28 days (AUC_0–28d_), to last measurable concentration (AUC_last_), and to time infinity (AUC_inf_). Samples were analyzed for serum PF‐03446962 concentrations at QPS (Newark, DE, USA) using a validated, sensitive, and specific chemiluminescence enzyme‐linked immunosorbent assay (ELISA). Serum specimens were stored at approximately −70°C until analysis. Calibration standard responses were linear over the 100–2500 ng/mL range. The lower limit of quantification for PF‐03446962 was 100 ng/mL. The between‐day assay accuracy, expressed as percent relative error for quality control concentrations, ranged from −5.7% to 9.6% for the quality control samples.

Clearance, *t*_1/2_, and volume of distribution at steady state for PF‐03446962 were estimated using noncompartmental analysis. Blood samples for PK assessments were collected predose; on days 1, 3, 5, 8, 11, 15, and 22 of cycle 1; and on day 1 of the following cycles. Monthly samples were collected up to 3 months after administration of the last dose of study drug.

#### Immunogenicity

Blood samples were collected to assess human anti‐human antibodies at each cycle prior to study drug dosing and monthly thereafter, up to 3 months after the last administered dose.

#### Pharmacodynamics

Pharmacodynamic evaluations were performed in patients treated with PF‐03446962 who had baseline and on‐treatment samples available for analysis. Blood samples were collected at screening, before and after infusion of PF‐03446962 on days 1 and 22 of cycle 1, on day 1 of cycles 2 and 3 prior to infusion, and at the end of the study. Changes in serum concentrations of ALK‐1 signaling and angiogenesis‐related proteins were measured by Aushon BioSystems (Billerica, MA) using a validated analytical assay. Angiopoietin 2, BMP‐9, endoglin, intercellular adhesion molecule 1, monocyte chemotactic protein, placental growth factor (PLGF), TGF‐*β*1, vascular cell adhesion protein, VEGF‐A, VEGF‐C, VEGF‐D, and VEGFR 1, 2, and 3 were included in the biomarker analysis.

#### Antitumor activity

Assessments were performed by computed tomography or MRI of the chest, abdomen, and pelvis at screening, every 6 weeks during treatment, and at the end of treatment or study withdrawal. Objective response was determined by Response Evaluation Criteria in Solid Tumors, version 1.1. Complete response and partial response were confirmed by computed tomography or MRI at least 4 weeks after first documentation. Clinical benefit rate was defined as confirmed complete response + confirmed partial response + stable disease (SD) lasting for at least 12 weeks (84 days) after the first dose.

### Statistical analysis

At least three and up to six patients were to be enrolled at each dose level in Part 1 to determine dose‐limiting toxicity (DLT) following a standard 3 + 3 study design and a total of six to ten patients at each of the two dose levels selected for Part 2 of the study. Descriptive statistics were used throughout the study. A two‐sided 95% confidence interval (CI) was calculated for overall response and for median PFS.

## Results

### Patients and treatment

A total of 36 patients were enrolled in the study. Sixteen patients received treatment with PF‐03446962 in Part 1 (*n *=* *4 treated with 4.5 mg/kg, *n *=* *3 with 7 mg/kg, and *n *=* *9 with 10 mg/kg) and 20 patients in Part 2 (*n *=* *10 each at the 7‐ and 10‐mg/kg dose levels). Of the nine patients with HCC on study, four received PF‐03446962 7 mg/kg and five received 10 mg/kg.

Patient demographics and clinical characteristics are presented in Table 1 for all patients on study. The majority had stage IV disease (*n *=* *32; 89%) and Eastern Cooperative Oncology Group performance status (ECOG PS) 0 (64%). Fifteen patients had colorectal cancer and nine had HCC. Other tumor types included gastric cancer, non–small‐cell lung cancer, renal cell carcinoma, gastrointestinal stromal tumor (GIST), soft tissue sarcoma, malignant neoplasia of the ampulla of Vater, and cancer of the appendix.

**Table 1:** Patient baseline demographics and clinical characteristics

	PF‐03446962 dose						
	4.5 mg/kg*n *=* *4		7 mg/kg*n *=* *13		10 mg/kg*n *=* *19		
	Male	Female	Male	Female	Male	Female	Total *N *=* *36
No. of patients	3	1	8	5	14	5	36
Age, years							
Median	64.0	49.0	56.0	53.0	60.0	56.0	56.5
Range	51–72	–	41–79	37–62	29–75	55–66	29–79
Race, *n*							
Japanese	2	1	3	2	9	2	19
Korean	1	0	5	3	5	3	17
ECOG PS, *n* (%)							
0	3 (75.0)		7 (53.8)		13 (68.4)		23 (63.9)
1	1 (25.0)		6 (46.2)		6 (31.6)		13 (36.1)
Prior surgeries, *n* (%)							
No	1 (25.0)		2 (15.4)		3 (15.8)		6 (16.7)
Yes	3 (75.0)		11 (84.6)		16 (84.2)		30 (83.3)
Prior radiation therapy, *n* (%)							
No	3 (75.0)		9 (69.2)		17 (89.5)		29 (80.6)
Yes	1 (25.0)		4 (30.8)		2 (10.5)		7 (19.4)
Prior non‐VEGFR‐targeted systemic therapies, *n* (%)							
No	0		1 (7.7)		2 (10.5)		3 (8.3)
Yes	4 (100)		12 (92.3)		17 (89.5)		33 (91.7)
Prior VEGFR‐targeted systemic therapies, *n* (%)							
No	0		1 (7.7)		6 (31.6)		7 (19.4)
Yes	4 (100)		12 (92.3)		13 (68.4)		29 (80.6)

ECOG PS, Eastern Cooperative Oncology Group performance score; VEGFR, vascular endothelial growth factor receptor.

All patients with HCC (*n *=* *9) were classified as Child Pugh class A. Eight of these patients had extrahepatic disease dissemination and four had vascular infiltration in hepatic vessels. Six patients with HCC had hepatic cirrhosis, three had a history of hepatitis B, four of hepatitis C, and two of alcohol abuse. None had nonalcoholic steatohepatitis or portal vein thrombosis.

Among all patients on study, the majority had received prior surgery (83%), prior non‐VEGFR‐targeted systemic anticancer treatment (92%), and prior VEGFR‐targeted therapy (81%). Only a minority of patients (19%) had been previously treated with radiation therapy (Table 1).

### MTD and safety

No DLTs were noted in the 12 DLT‐evaluable patients during the dose escalation part of the study and the MTD for biweekly i.v. administration of PF‐03446962 in Asian patients was determined to be 10 mg/kg. Thirty‐five (97.2%) patients experienced at least one treatment‐emergent, all‐causality AE. The most frequent all‐causality AEs (observed in more than four patients) included pyrexia (36.1%), thrombocytopenia (27.8%), constipation (13.9%), proteinuria (13.9%), and infection of the upper respiratory tract (13.9%) (Table S1). Two grades 4–5 AEs were observed in this study (both in the 7 mg/kg group); one death due to pneumonia and one grade 4 AE of increased lipase. Both AEs were deemed not to be treatment related. In the first case, a patient with renal cell carcinoma gradually worsened and the underlying disease was considered by the investigator as the most likely explanation of the clinical course leading to the patient's demise. In the second case, the grade 4 AE of increased lipase was considered disease related rather than treatment related, as this AE, usually seen in patients with periampullary cancers, was observed in a patient with a malignant neoplasm of the ampulla of Vater who had experienced an episode of lipase elevation prior to treatment. A total of 26 (72.2%) patients experienced treatment‐related AEs, of which the most frequent were thrombocytopenia (*n *=* *10, 27.8%), pyrexia (*n *=* *8; 22.2%), epistaxis (*n *=* *4, 11.1%), and telangiectasia (*n *=* *4, 11.1%) (Table 2). The majority of the treatment‐related AEs were mild to moderate in severity. Five patients experienced treatment‐related grades 1–2 thrombocytopenia and five patients had grade 3 thrombocytopenia. Incidence and severity of treatment‐related AEs reported for two or more patients in each dose group are presented in Table 3.

**Table 2:** All‐grade, treatment‐related adverse events reported in >10% patients

*N *=* *36	All Grades *n* (%)	Grade 1 *n (*%)	Grade 2 *n* (%)	Grade 3 *n* (%)	Grade 4 *n* (%)
Any AE^a^	26 (72.2)	12 (33.3)	6 (16.7)	8 (22.2)	0 (0.0)
Thrombocytopenia^b^	10 (27.8)	1 (2.8)	4 (11.1)	5 (13.9)	0 (0.0)
Pyrexia	8 (22.2)	7 (19.4)	1 (2.8)	0 (0.0)	0 (0.0)
Epistaxis	4 (11.1)	4 (11.1)	0 (0.0)	0 (0.0)	0 (0.0)
Telangiectasia	4 (11.1)	3 (8.3)	1 (2.8)	0 (0.0)	0 (0.0)

AE, adverse event.

a No grade 5 treatment‐related AEs were reported in this study.

b Includes thrombocytopenia and decreased platelet count.

**Table 3:** All‐grade, treatment‐related adverse events reported in two or more patients by dose level

	All Grades *n* (%)	Grade 1 *n (*%)	Grade 2 *n* (%)	Grade 3 *n* (%)	Grade 4 *n* (%)
Dose level: 4.5 mg/kg, (*N *=* *4)					
Any AE^a^	3 (75.0)	1 (25.0)	2 (50.0)	0 (0.0)	0 (0.0)
Proteinuria	2 (50.0)	0 (0.0)	2 (50.0)	0 (0.0)	0 (0.0)
Dose level: 7 mg/kg, *N *= (13)					
Any AE^a^	9 (69.2)	4 (30.8)	1 (7.7)	4 (30.8)	0 (0.0)
Thrombocytopenia^b^	4 (30.8)	0 (0.0)	1 (7.7)	3 (23.1)	0 (0.0)
Conjunctival hemorrhage	3 (23.1)	3 (23.1)	0 (0.0)	0 (0.0)	0 (0.0)
Epistaxis	3 (23.1)	3 (23.1)	0 (0.0)	0 (0.0)	0 (0.0)
Pyrexia	3 (23.1)	3 (23.1)	0 (0.0)	0 (0.0)	0 (0.0)
Telangectasia	3 (23.1)	2 (15.4)	1 (7.7)	0 (0.0)	0 (0.0)
Dose level: 10 mg/kg, (*N *=* *19)					
Any AE^a^	14 (73.7)	7 (36.8)	3 (15.8)	4 (21.1)	0 (0.0)
Thrombocytopenia^b^	6 (31.6)	1 (5.3)	3 (15.8)	2 (10.5)	0 (0.0)
Pyrexia	5 (26.3)	4 (21.1)	1 (5.3)	0 (0.0)	0 (0.0)
Constipation	2 (10.5)	2 (10.5)	0 (0.0)	0 (0.0)	0 (0.0)
Decreased appetite	2 (10.5)	2 (10.5)	0 (0.0)	0 (0.0)	0 (0.0)
Headache	2 (10.5)	2 (10.5)	0 (0.0)	0 (0.0)	0 (0.0)
Vomiting	2 (10.5)	2 (10.5)	0 (0.0)	0 (0.0)	0 (0.0)

AE, adverse event.

a No grade 5 treatment‐related AEs were reported in this study.

b Includes thrombocytopenia and decreased platelet count.

**Figure 1:**
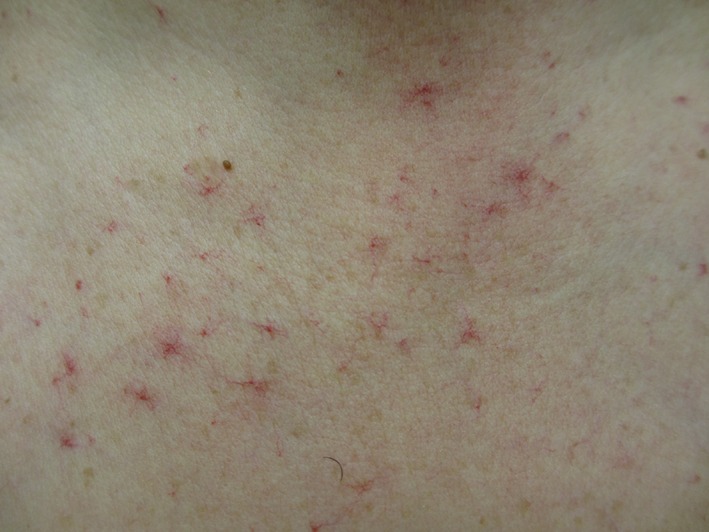
Example of telangiectasia observed in a patient with hepatocellular carcinoma treated with PF‐03446962.

Four (11.1%) patients developed grade 1 (*n *=* *3; one patient in the 4.5 and two patients in the 7 mg/kg group) or grade 2 (*n *=* *1; 7 mg/kg group) telangiectasia (e.g., on chest, back, neck, arms, and legs)—among them, three patients with HCC and one patient with colorectal cancer. An example of telangiectasia, observed in a patient with stage IV HCC and SD lasting at least 84 days following treatment with PF‐03446962 7 mg/kg, is shown in Figure 1.

**Figure 2:**
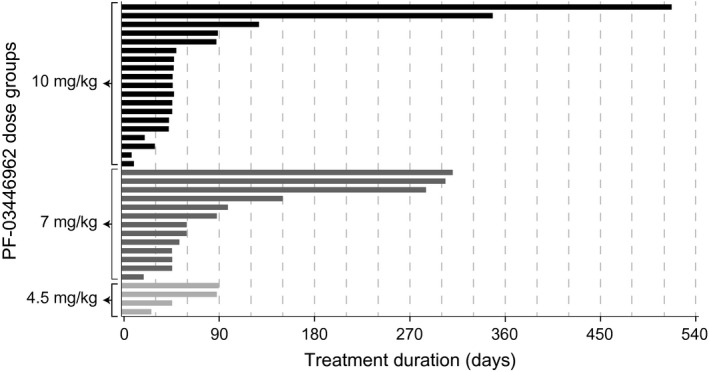
Duration of treatment with PF‐03446962 across dose levels.

Treatment was permanently discontinued or the dose reduced because of a treatment‐related AE in one patient each. One 79‐year‐old man (7 mg/kg group) experienced treatment‐related interstitial pneumonia with exertional dyspnea, which resolved after treatment discontinuation. A 75‐year‐old man (10 mg/kg group) developed grade 3 thrombocytopenia that resolved following dose reduction of study drug. Temporary discontinuations occurred in four patients for an all‐cause AE, including grade 2 viral infection, grade 3 hyperkalemia, grade 3 increased lipase, and grade 3 increased aspartate aminotransferase (*n *=* *1 each). Treatment was temporarily discontinued in four patients for a treatment‐related AE, including asymptomatic grade 3 increased amylase (*n* = 1; 7 mg group), grade 3 thrombocytopenia (*n* = 2; 7 mg group), and grade 2 thrombocytopenia (*n* = 1, 10 mg group). All of these treatment‐related AEs resolved upon temporary treatment discontinuation. The median duration of treatment (from first dose to discontinuation date) was 45 days (range, 5–515 days) (Fig. 2) and the median number of cycles was 2 (range, 1–30).

### Pharmacokinetics

**Figure 3:**
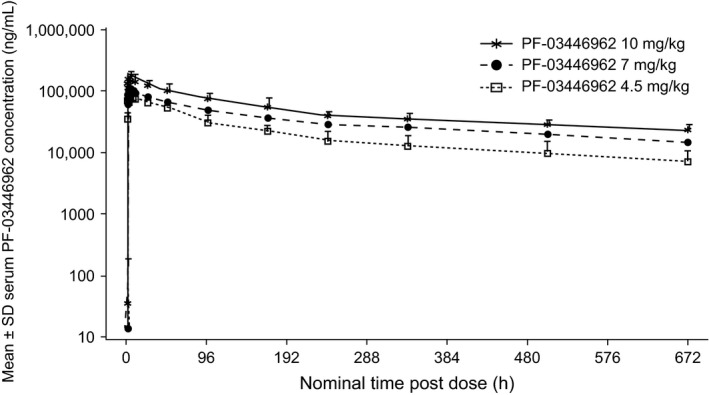
Mean serum PF‐03446962 concentration–time profiles following single‐dose administration in cycle 1.

PF‐03446962 showed a biphasic PK profile following single‐dose i.v. infusion (Fig. 3). The median *T*_max_ values were 1.5–2 h, and the mean *t*½ was 14.2 days in the 7 mg/kg dose group (Table 4). PF‐03446962 exposure appeared to be similar across all doses, based on dose‐normalized geometric mean AUC values, including AUC_0–28d_, AUC_last_, AUC_inf_, and maximum concentration. Overall, PF‐03446962 exposure increased in an approximately dose‐proportional manner across the 4.5–10 mg/kg dose range (Table 4).

**Table 4:** Summary of PF‐03446962 pharmacokinetic parameters

Parameter, Units^a^	PF‐03446962		
	4.5 mg/kg	7 mg/kg	10 mg/kg
*N*	4	13^c^	19^d^
AUC_0–28d_, ng h/mL	12,360,000 (36)	21,980,000 (14)^c^	30,360,000 (22)^d^
AUC_0–28d_(dn), ng h/mL/mg	42,400 (27)	51,790 (18)^c^	46,940 (20)^d^
AUC_inf_, ng h/mL	15,500,000 (40)	27,630,000 (13)	29,300,000, 46,200,000^b^
AUC_inf_(dn), ng h/mL/mg	53,170 (31)	67,460 (20)	57,700, 58,700^b^
AUC_last_, ng h/mL	12,050,000 (39)	20,500,000 (30)	26,070,000 (62)
AUC_last_(dn), ng h/mL/mg	41,300 (29)	48,770 (29)	40,230 (55)
*C*_max_, ng/mL	89,420 (47)	121,100 (34)	169,000 (28)
*T*_max_, h	1.74 (0.983–8.00)	2.00 (0.900–8.02)	1.50 (0.950–8.07)
*C*_trough_, ng/mL	6689 (47)	14,680 (22)^c^	22,940 (27)^d^
*t*_½_, days	14.58 ± 2.21	14.16 ± 1.78	15.6, 17.4^b^
CL, L/h	0.01880 (31)	0.01482 (20)	0.0170, 0.0173^b^
*V*_ss_, L	7.535 (23)	6.556 (23)	7.60, 9.08^b^

a Unless otherwise specified, summary statistics are geometric mean (%CV) for all parameters except: median (range) for *T*_max_; arithmetic mean (±standard deviation) for t_½_.

b Individual values presented for less than three patients. Only patient pharmacokinetic profiles with a well characterized terminal phase (defined by at least three data points, *r* ^*2*^ ≥ 0.9, and AUC_extrap_ ≤30%) are included in this analysis.

c *n *=* *12 (not reported for one patient with incomplete data).

d *n *=* *14 (not reported for five patients with incomplete data).

AUC_0–28d_, area under the serum concentration–time curve from time zero to 28 days; AUC_extrap_, area under the concentration–time curve extrapolation; AUC_inf_, area under the serum concentration–time curve from time zero to infinity; AUC_last_, area under the serum concentration–time curve from time zero to last measureable time point; CL, clearance; *C*_max_, maximum serum concentration; *C*_trough_, lowest serum concentration before the next dose; dn, dose normalized to a 1‐mg dose; *T*_max_, time to maximum serum concentration; *V*_ss_, volume at steady state.

### Antitumor activity

No objective responses were observed in this study (Table S2). Nine (25.7%) of the 35 evaluable patients had clinical benefit with SD for ≥84 days, across dose levels (range, 22.2–30.8%) and tumor diagnosis, including HCC, colorectal cancer, non–small‐cell lung cancer, renal cell carcinoma, and GIST. Changes in tumor diameter from baseline in PF‐03446962‐treated patients are shown in Figure S1.

Four (44.4%) of the nine patients with HCC had SD for ≥84 days and three of these patients also experienced telangiectasia. Of note, SD lasting from 247 to 417 days was noted in patients with HCC (*n *=* *2), renal cell carcinoma (*n *=* *1), or GIST (*n *=* *1) who had progressed following prior VEGF‐targeted, antiangiogenesis therapy.

The median PFS was 1.4 months (95% CI: 1.3–2.7) in all patients (*n *=* *35) and 1.8 months (95% CI: 0.9–9.2) in patients with HCC (*n *=* *9).

### Pharmacodynamics

**Figure 4:**
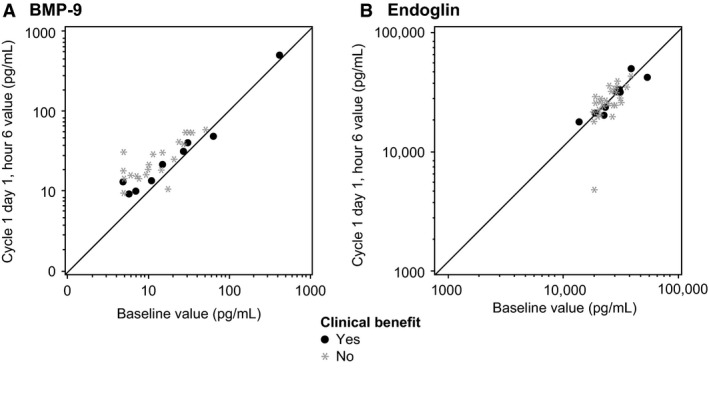
Individual circulating levels of (A) bone morphogenetic proteins (BMP)‐9 and (B) soluble endoglin detected in patients at baseline and 6 h postinfusion of PF‐03446962 (day 1, cycle 1), according to treatment response. Clinical benefit includes complete or partial responses and stable disease lasting for ≥84 days.

Mean levels of PLGF decreased from baseline by 7% at day 1 of cycle 2 and by 22% at the end of PF‐03446962 treatment (*n *=* *36). No clear association was noted between PLGF blood levels and treatment benefit. No other consistent trends and correlations with clinical endpoints were observed in the biomarkers related to ALK‐1 and the angiogenesis signaling pathways evaluated in this study, including circulating levels of BMP‐9 and soluble endoglin as shown in Figure 4. Baseline and posttreatment levels of BMP‐9 and soluble endoglin are presented for each patient according to clinical benefit experienced following treatment with PF‐03446962, including complete or partial responses and SD lasting for ≥84 days.

## Discussion

We report here the results of the first dose‐finding study conducted with the fully human anti‐ALK‐1 mAb PF‐03446962 in Asian patients with advanced solid malignancies. PF‐03446962 was well tolerated in this patient population and the MTD for biweekly i.v. administration was estimated to be 10 mg/kg, as previously determined in a Western population of patients with solid tumors treated with PF‐03446962 16, 17. No human antihuman antibodies were detected during treatment with PF‐03446962 in this study (data not shown). The PK characteristics observed following i.v. administration of PF‐03446962 in this study were comparable to those reported in Western patients 16, 17. Median serum concentrations remained above the estimated effective concentration for 14 days following single‐dose administration of PF‐03446962.

Five (13.9%) patients experienced treatment‐related grades 1–2 thrombocytopenia and five (13.9%) developed treatment‐related grade 3 thrombocytopenia. The most frequent nonhematologic treatment‐related AEs observed in this study were grades 1–2 pyrexia (22.1%) and grade 1 epistaxis (11.1%). Grades 1–2 telangiectasia was reported in 11.1% of patients. The development of telangiectasia in four patients in this study (three with a primary diagnosis of HCC) suggests vascular targeting and in vivo inhibition of the ALK‐1 pathway by PF‐03446962, consistent with the genetically determined loss of ALK‐1 functions reported in patients with type 2 HHT 7, 8, 9. Treatment with PF‐03446962 was also associated with a reduction in the circulating levels of PLGF at the end of treatment, compared with baseline. Reductions in PLGF have been reported to decrease neovascularization, hepatic recruitment, and hypoxia in a mouse model of HCC 18. Further investigations will help to identify biomarkers for prediction of response to PF‐03446962.

Preliminary evidence of clinical activity was noted in this study following treatment with single‐agent PF‐03446962. SD for ≥84 days was observed in 25.7% of patients across dose levels and in 44.4% of patients with HCC. Importantly, four patients with HCC, renal cell carcinoma, or GIST, who had previously received anti‐VEGF therapy and experienced disease progression, achieved SD lasting more than 6 months.

Antitumor activity has been observed with other investigational ALK‐1/endoglin complex targeting agents, such as the activin receptor‐like kinase‐1 ligand trap dalantercept and the anti‐endoglin (CD105) antibody TRC105, in patients with solid tumors 20, 21, 22, 23, 24. One (2.7%) patient with squamous cell cancer of the head and neck achieved a partial response and eight (21.6%) patients had stable disease lasting 12 weeks or more in a phase Ib study of dalantercept conducted in 37 patients with advanced solid tumors 20. Conversely, insufficient clinical activity was noted with dalantercept in 28 patients with recurrent/persistent endometrial cancer 21. Furthermore, a grade 5 event due to gastric hemorrhage potentially related to dalantarcept treatment was reported in this phase II study, in an endometrial cancer patient with radiation fibrosis and small bowel obstruction 21. Administration of the anti‐endoglin antibody TRC 105 as a single agent was reported associated with a reduction in PSA levels in eight (40%) of 20 patients with metastatic castration‐resistant prostate cancer 22, and with one (9.1%) partial response among 11 patients with advanced HCC and disease progression following prior sorafenib therapy 23. In addition, treatment with a combination of TRC105 and bevacizumab in 38 patients with advanced tumors induced a partial response in two (5.3%) patients and lasting stable disease in six (15.8%) patients 24.

Based on the safety, tolerability, and PK findings of this study, the RP2D for further clinical evaluation of PF‐03446962 in adult Asian patients with advanced malignancies was determined to be 7 or 10 mg/kg i.v. administered every 2 weeks. The 7 mg/kg dose appeared more appropriate for patients with HCC, in view of the lower effect on platelet cell count observed at this dose level in this patient population. Both the 7 and 10 mg/kg doses exceed the efficacious concentration estimated in preclinical studies for PF‐03446962.

PF‐03446962‐mediated inhibition of ALK1 signaling was expected to disrupt tumor angiogenesis and inhibit tumor growth when administered as monotherapy, based on the results obtained in preclinical studies 15. Consistent with this hypothesis, treatment with single‐agent PF‐03446962 demonstrated preliminary antitumor activity in patients with solid malignancies, and particularly HCC, as reported in this study. Combination of PF‐03446962 with other agents, such as sorafenib, may allow targeting of multiple phases of the angiogenic process associated with tumor growth and thus potentially provide increased benefit to patients.

In conclusion, PF‐03446962 represents a novel strategy to block angiogenesis that may be complementary to current treatment with anti‐VEGF agents, VEGFR kinase inhibitors, or chemotherapy in patients with solid malignancies.

## Conflict of Interest

Drs. Y. J. Bang and M. Ikeda received research funding from Pfizer. Drs. T. Hirohashi, C. Gallo Stampino, A. Suzuki, Y. Fujii, and J. A. Williams were full‐time employees of Pfizer during the conduct of this study. Drs. Gallo Stampino and J. A. Williams own stocks from Pfizer. Medical writing support was provided by Dr. S. Mariani of Engage Scientific Solutions and was funded by Pfizer. All authors had full access to all the data in the study and had final responsibility for the decision to submit for publication. Drs. T. Doi, K. Yoh, TY. Kim, TM. Kim, KH. Lee, and A. Ohtsu have no conflict of interest.

## Supporting information


**Figure S1.** Changes in tumor diameter from baseline in patients treated with PF‐03446962. Asterisks indicate prior treatment with VEGFR‐targeted inhibitors. VEGFR, vascular endothelial growth factor receptor.
**Table S1.** Treatment‐emergent, all‐causality, all‐grade adverse events in >10% of patients.
**Table S2.** Clinical activity in patients treated with PF‐03446962.
