# Supplementary material for: A phase I study of the human anti‐activin receptor‐like kinase 1 antibody PF‐03446962 in Asian patients with advanced solid tumors
Source: Cancer Med. 2016 Apr 14;5(7):1454–63. doi: 10.1002/cam4.724 (PMC4944871; doi:10.1002/cam4.724)

**ALK-1 1004 Asian Study Manuscript - Supporting Information**

**Supporting Table S1.** Treatment-emergent, all-causality, all-grade adverse events in >10% of patients*

| **Adverse Event** | **All Grades** | | **Grade 1** | | **Grade 2** | | **Grade 3** | | **Grade 4** | |
| --- | --- | --- | --- | --- | --- | --- | --- | --- | --- | --- |
|  | *n* | (%) | *n* | (%) | *n* | (%) | *n* | (%) | *n* | (%) |
| Any adverse event | 35 | (97.2) | 9 | (25.0) | 10 | (27.8) | 14 | (38.9) | 1 | (2.8) |
| Pyrexia | 13 | (36.1) | 12 | (33.3) | 1 | (2.8) | 0 | 0.0 | 0 | 0.0 |
| Thrombocytopenia^†^ | 10 | (27.8) | 1 | (2.8) | 4 | (11.1) | 5 | (13.9) | 0 | 0.0 |
| Constipation | 5 | (13.9) | 5 | (13.9) | 0 | 0.0 | 0 | 0.0 | 0 | 0.0 |
| Proteinuria | 5 | (13.9) | 1 | (2.8) | 4 | (11.1) | 0 | 0.0 | 0 | 0.0 |
| Upper respiratory tract infection | 5 | (13.9) | 0 | 0.0 | 5 | (13.9) | 0 | 0.0 | 0 | 0.0 |
| Blood alkaline phosphatase increased | 4 | (11.1) | 0 | 0.0 | 3 | (8.3) | 1 | (2.8) | 0 | 0.0 |
| Conjunctival hemorrhage | 4 | (11.1) | 4 | (11.1) | 0 | 0.0 | 0 | 0.0 | 0 | 0.0 |
| Epistaxis | 4 | (11.1) | 4 | (11.1) | 0 | 0.0 | 0 | 0.0 | 0 | 0.0 |
| Fatigue | 4 | (11.1) | 4 | (11.1) | 0 | 0.0 | 0 | 0.0 | 0 | 0.0 |
| Flank pain | 4 | (11.1) | 4 | (11.1) | 0 | 0.0 | 0 | 0.0 | 0 | 0.0 |
| Hemoptysis | 4 | (11.1) | 2 | (5.6) | 2 | (5.6) | 0 | 0.0 | 0 | 0.0 |
| Telangiectasia | 4 | (11.1) | 3 | (8.3) | 1 | (2.8) | 0 | 0.0 | 0 | 0.0 |

*One patient died of pneumonia (grade 5 AE not considered to be treatment-related).

^†^ Includes thrombocytopenia and decreased platelet count.

**Supporting Table S2.** Clinical activity in patients treated with PF-03446962

|  | PF‑03446962, *n* (%) | | | |
| --- | --- | --- | --- | --- |
| Parameter | 4.5 mg/kg *n* = 4 | 7 mg/kg *n* = 13 | 10 mg/kg *n* = 18 | **Total *n* = 35** |
| Complete response | 0 | 0 | 0 | 0 |
| Partial response | 0 | 0 | 0 | 0 |
| Stable disease ≥ 84 days | 1 (25.0) | 4 (30.8) | 4 (22.2) | 9 (25.7) |
| Stable disease < 84 days | 0 | 0 | 0 | 0 |
| Objective progression | 2 (50.0) | 7 (53.8) | 13 (72.2) | 22 (62.9) |
| Symptomatic deterioration | 0 | 0 | 1 (5.6) | 1 (2.9) |
| Early death | 0 | 1 (7.7) | 0 | 1 ( 2.9) |
| Indeterminate | 1 (25.0) | 1 (7.7) | 0 | 2 ( 5.7) |
| Clinical benefit response rate (complete+partial response+stable disease*) | 1 (25.0) | 4 (30.8) | 4 (22.2) | 9 (25.7) |
| 95% exact CI | 0.6–80.6 | 9.1–61.4 | 6.4–47.6 | 12.5–43.3 |

*Stable disease lasting for at least 84 days after first dose of study drug.

CI, confidence interval.


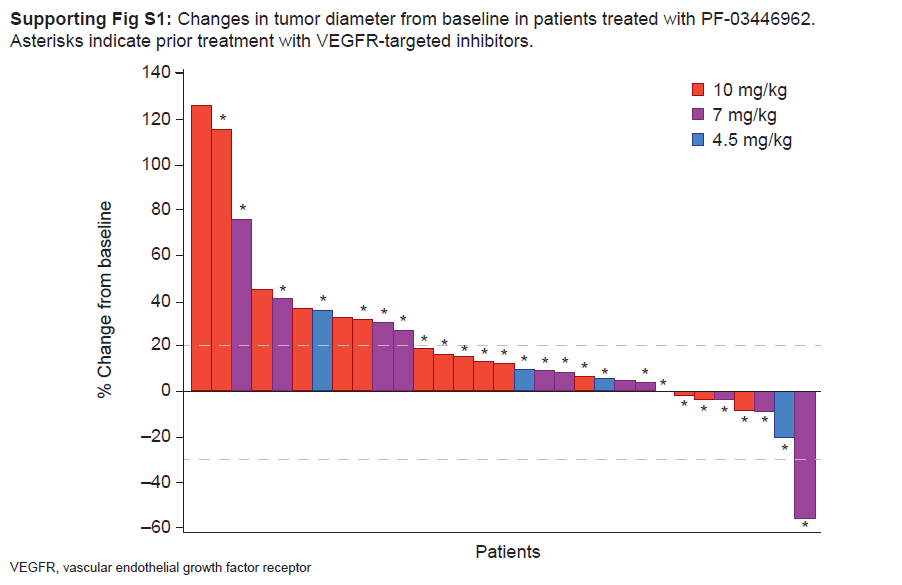

Supplement: Supplementary file 1 — Figure S1. Changes in tumor diameter from baseline in patients treated with PF‐03446962. Asterisks indicate prior treatment with VEGFR‐targeted inhibitors. VEGFR, vascular endothelial growth factor receptor. Table S1. Treatment‐emergent, all‐causality, all‐grade adverse events in >10% of patients. Table S2. Clinical activity in patients treated with PF‐03446962. [file CAM4-5-1454-s001.docx]
